# Robotic Integration in the Field of Opthalmology and Its Prospects in India

**DOI:** 10.7759/cureus.30482

**Published:** 2022-10-19

**Authors:** Bhawna Kumari, Pravin Tidake

**Affiliations:** 1 Ophthalmology, Jawaharlal Nehru Medical College, Datta Meghe Institute of Medical Sciences, Wardha, IND; 2 Ophthalmology, Jawarhalal Nehru Medical College, Datta Meghe Institute of Medical Sciences, Wardha, IND

**Keywords:** haptic feedback, microsurgery, tele-manipulation, viteroretinal surgery, robotics

## Abstract

In this paper, an overview of the integration of robotic techniques into surgical fields of ophthalmology is described and the details about the latest advancements and future potentials associated with it are presented.

The eye is a small, enclosed space that does not tolerate the misplacement of instruments that general surgery can tolerate. As the retina doesn't regenerate, it is of paramount importance to avoid injury. Furthermore, there are additional limitations of unassisted human hands in terms of dexterity, tremor, and precision in positioning instruments in the eye. Robotics has become a promising solution to these human challenges. The emergence of robotic technology into the domain of rapidly advancing micro-invasive surgery has reduced discomfort in patients and enhanced safety, capabilities, and outcomes. With the arrival of the Femtosecond laser system for robotic cataract surgery in several hospitals in India, the paradigm of robotic surgery has shifted as people started to accept and apply it. Although there is still much to learn in this area, there is growing interest in creating gadgets that perform complete surgical procedures. The fundamental objective of these surgeries would be to increase speed and efficiency without compromising the capacity to increase precision. Major criteria include an acceptable range of motion, the capacity to switch instruments mid-surgery, and simultaneous manipulation of the surgical instrument.

Robotic surgery is an already well-established technological advancement employed across the globe by leading surgeons in their fields but its curve in ophthalmology is still under supervision. Just like every other advance, robotics has its own set of disadvantages including but not limited to the costs, limited availability, and long learning curve. Nonetheless, this paper doesn't intend to promote the replacement of surgeons with technology, it's intended to get aware of the utilities of technology to improve care and deliver personal compassionate care. This quest is for the idea of robotics in the ocular field and improvisation of the field.

## Introduction and background

A robot in literal meaning is a machine that is designed to perform tasks that are unintelligible, repetitive, and those tasks which can’t be possibly done by the human workforce. Robot-assisted surgery is an innovative type of surgical technique that uses robotic technologies. In the medical specialty of ophthalmology, there has been an improvisation in clinical care and the assistance offered in terms of three-dimensional views, superior instrument maneuverability, increased magnification, and diminished error possibility [1]. A surgical robot is a sophisticated instrument with advanced sensing and display capabilities that are computer-controlled or assisted. It could have surgical operations motion-programmed into it. Stating the facts, robotic technology is able to provide a precision of 1 mm currently [2]. To execute a delicately accurate neurosurgical operation, the first robotic surgery was undertaken in 1985. The first robotic laparoscopic cholecystectomy was completed in 1987 [3]. The limitations of conventional "open" surgical procedures have spurred revolutions in modern surgical techniques. Laparoscopic and robotic surgery has become more popular, and general surgery has evolved in favor of less invasive methods.

The ocular field has been improved by the integration of robotic surgical intervention into operating rooms. Robotic surgery is currently advancing quickly, particularly in the field of minimally invasive surgery (MIS) [4]. The possibilities and uses of robotic eye surgery have developed tremendously over the past ten years. It is a rapidly developing technology. Improved accuracy, better usability, better visualization, and improved depth resolution have increased both the safety and efficacy of the procedures. Benefits also include safer surgery, minimizing visible skin incisions, faster recovery times, faster and more accurate surgery, and postoperative pain relief. However, their implementation in ocular surgeries has trailed behind compared to other surgical fields.

Manually performing a surgery, especially intraocular microsurgery is technically challenging to perform despite the advancements in the instruments. Ranging from an intrinsic hand tremor or difficulty in holding a surgical instrument stationary for prolonged durations to the limited resolution of the human eye and precision in positioning instruments in an organ as small as the eye [5]. These limitations make humans insufficient and thus come to light the robots with integrated imaging modalities such as digital microscopy and optical coherence tomography (OCT). Procedures such as maintaining cannulation and inserting a retinal vein cannula with successful infusion are technically beyond the scope of the majority of surgeons [6]. In addition, the challenges faced by surgeons in determining the optimal timing of a procedure, and the optimal drug for administration, collectively limit the feasibility of the procedure.

Recent progress in India with the introduction of Femtosecond laser surgery or blade-free cataract surgery for cataract patients can be seen as a new era of beginnings in a developing country like India. A significant scope can be seen in the future of robotics implementation in ophthalmology where it can prove to be filling the gaps left by a lack of professional assistance. There are several robotics technologies that have been successfully integrated into ocular surgeries. This article gives an overview of the technologies and their potential.

## Review

Robotics in ophthalmology

In the medical discipline of ophthalmology, steady hand movements, appropriate lighting, and an unhindered, unobstructed view are essential. Although it has a lot of potential, robotic equipment deployment in the field of optics is still in its infancy. One of the most technically challenging minimally invasive surgical techniques in this area is vitreoretinal microsurgery [1]. The retina presents micron-scale objectives in a close or tight workspace that require handling with remarkable precision in a small, delicate workspace. Therefore, many of the problems related to ocular microsurgery can be addressed logically, strategically, and hopefully thanks to the special qualities of the robotic approach. The anterior segment of the eye is also being examined when it comes to robotic integration in ophthalmic surgery, in addition to the retina. The robotic devices can be used for mechanical trephination, cardinal sutures, continuous 10/0 nylon sutures, and suture modifications. Surgical robotic systems are divided into four groups based on how they interact with the surgeon: 1. Handheld, 2. tale manipulated or remote control 3. hand-on-hand, and 4. magnetically operated.

High-precision robotics are required in the anatomically restricted environment of the ocular field. This led to solutions that were based on one of these three strategies. The first is a direct support strategy that stabilizes the physiological tremor of handheld instruments, thereby enhancing the surgeon's dexterity. A second approach is co-manipulation, where a robotic system is designed to work with the surgeon to move the instrument giving the surgeon the freedom to adjust the resistance and movement needed to perform the intervention. In such a collaborative robotic system, the surgeon performs the surgery by directly holding the surgical instrument connected to the actuator. The movement of the device is mainly determined by the surgeon, as the actuators release assistive forces to reduce hand tremors and limit movement within a safe range. A third approach is telemanipulation, in which the robot's micromanipulator is called through the master-slave system to properly perform a series of tasks and assist the surgeon's actions as needed. The auxiliary device reproduces the (usually reduced) motion produced by the operator with the main unit with higher operational accuracy based on haptic feedback. Haptic feedback involves information and control related to the human sense of touch. The lack of haptic feedback to the surgeon regarding the force of interaction between the tooltip and the target tissue is a major limiting factor without which the surgeon would lose the ability to palpate for evaluating variations in tissue stiffness which can be done during open surgery. Especially in complex surgical tasks such as tissue suturing or knot-tying that require greater precision.

The Da Vinci Surgical System

It has befitted itself as the most prevalent robotic surgical system with facilities installed around the world. The robotic system's optical magnification and capacity to filter and manage vibrations are its most crucial features [2]. The system is created in a particular way that simplifies the movement due to the presence of joints in the robot arm which has a 360-degree movement [3]. It can be utilised successfully in pterygium surgery, penetrating keratoplasty, anterior capsule, intraocular foreign body removal, and corneal tear repair [4,5]. The FDA has approved the commercially available robotic surgical system known as the Da Vinci Surgical System (Intuitive Surgical, Inc., Sunnyvale, USA). Later, Mimic and Intuitive Surgery Inc. launched the da Vinci Skill Simulator specifically designed to allow surgeons to enhance their skills before performing real surgeries.

Limitations of this system that came into knowledge were the presence of a high remote center of motion (RCM) in the robot, which is located directly on the wrist and a great distance from the head of the device. This leads to unnecessary stress and the need for another RCM in the process at the site of penetration [6]. In addition, the robot has a video recording system designed primarily for endoscopic use and cannot provide optimal and detailed images like that of an operating microscope [7].

**Figure 1:**
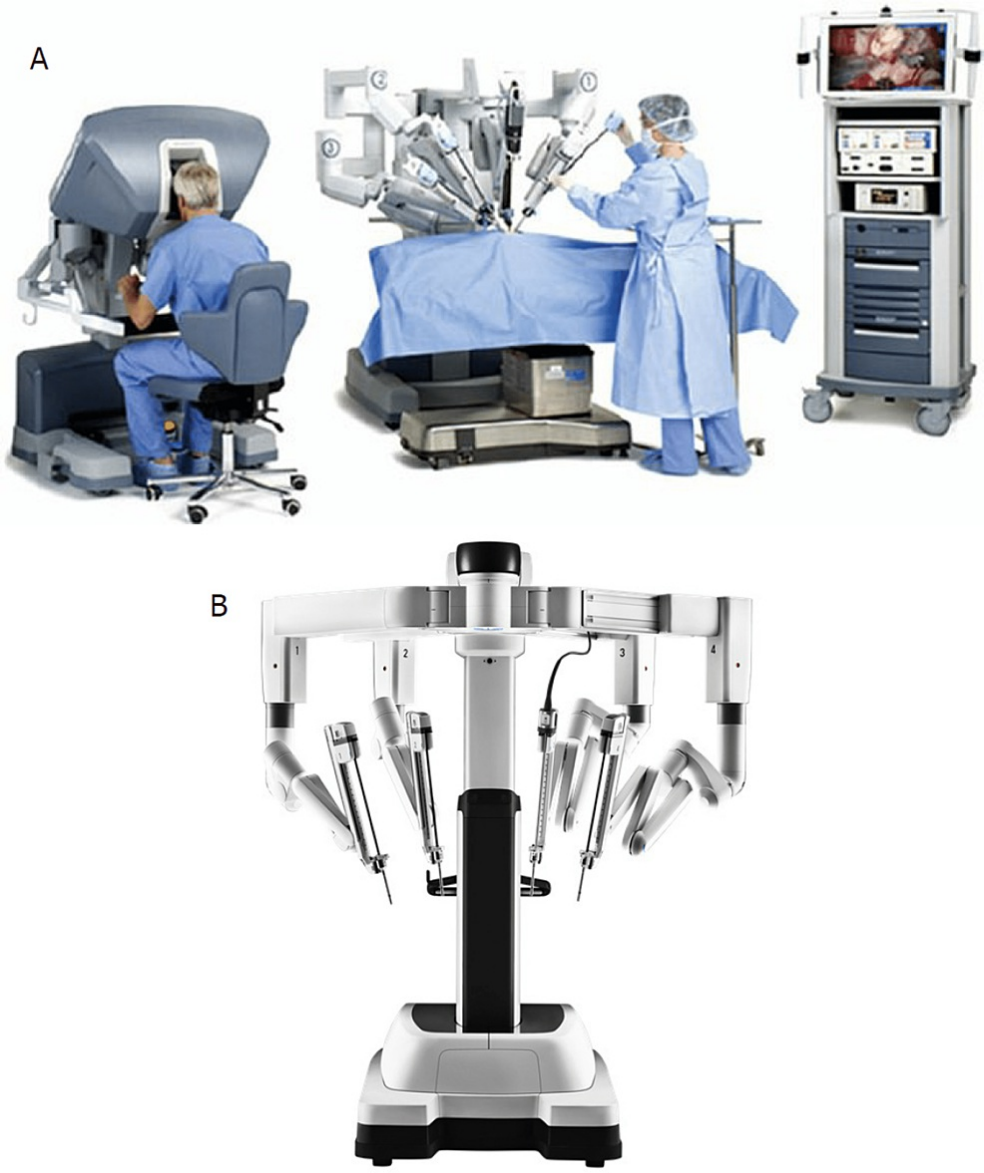
Model of the Da Vinci Surgical System having three major components: (1) the surgeon’s console; (2) a surgical cart with the robotic arms and end-effectors; and (3) the visual cart. This image is taken from an open-access journal licensed under a Creative Commons Attribution 4.0 International License (Gumbs et al. [9]).

Figure 1 shows the model of the Da Vinci Surgical System [8,9].

Force sensor

The force applied during surgery to the retina is often less than the surgeon's ability to detect. As a result, force-sensing devices are now considered cutting-edge technology. The apparatus that responds to forces and can record the forces used during a procedure [8]. The development of the force-sensing instruments is based on the concept of remote center of motion (RCM) in robot postulates - only three degrees of freedom (DOFs) rotate at the scleral entry point and one DOF translates along the axis of the device. While all lateral displacements are restricted at the point of entry by mechanical restrictions through the relatively rigid eye wall [10].

The Micron

It is an active handheld micromanipulator. Using an image guidance approach reduces hand tremor and tooltip positional error. It detects motion, uses sophisticated filtering algorithms to separate between desired (needed) and unwanted motion, and actively counteracts unwanted motion by deflecting its tip in the opposite direction [11-13].

**Figure 2:**
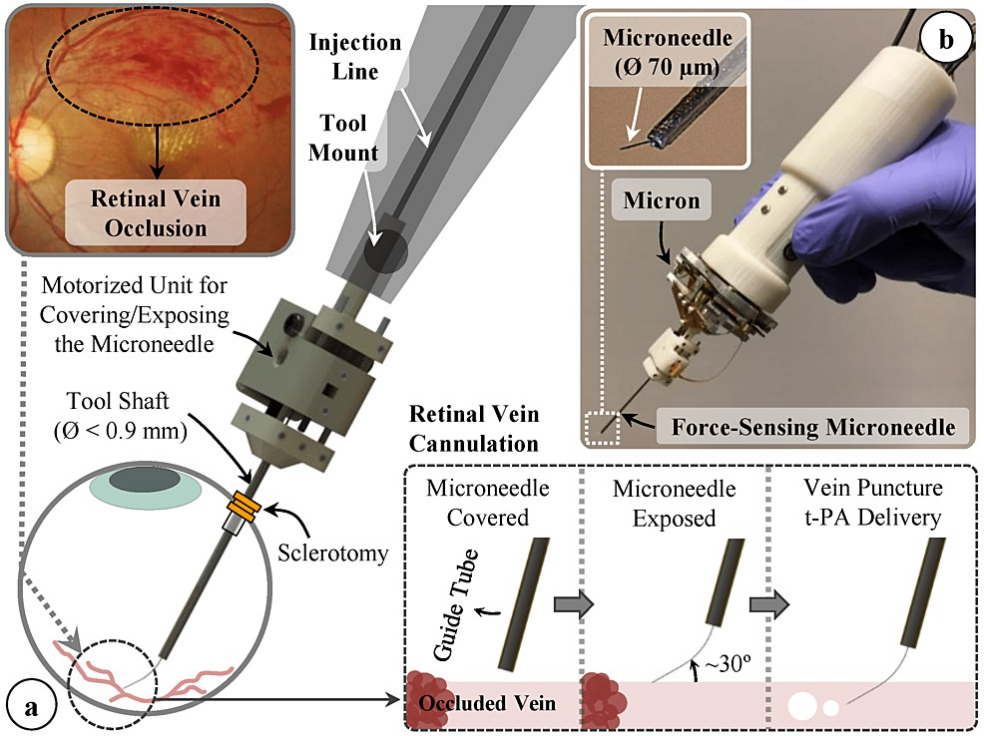
a) Conceptual design of our motorized force-sensing microneedle for retinal vein cannulation; (b) The tool has a thin tip (Ø 70 µm) and a modular design that allows for easy integration with robotic devices, such as the Micron handheld micromanipulator. This image has been taken from an open-access journal (Gonenc et al. [13]).

Figure 2 shows the conceptual design of our motorized force-sensing microneedle for retinal vein cannulation [13].

RAMIS - Robotic Arm Manipulators

It is a hybrid series-parallel combination incorporating piezoelectric motors for actuation. It consists of two parallel joints, one prismatic joint, and one optional rotary joint to allow 6 DOFs of tool movement with greater rigidity and better accuracy. It has a compact design that is attached to the patient's head to reduce the influence of the patient's movement [14]. It has been used by researchers to monitor sub-pinhole depth with OCT and to supplement microscopic imaging by displaying OCT-generated volumetric scans [15].

The Integrated Robotic Intraocular Snake or IRIS

It is a handheld or mountable robot (on a) surgical tool prototype that provides the surgeon with intraocular dexterity. It was originally designed as a combination of operating tools having 20-gauge sizings with an outside diameter of 0.9 mm [16]. The continuous segment of IRIS is 10 mm long and has 2 DOFs rotates each with ±45 degrees of flexibility [17]. Sadly, due to the low precision of the actuation resolution that limits the accuracy, this does not seem to work.

Preceyes Surgical System

It acts as a robotic assistant for vitreoretinal surgery. It assists surgeons by measuring and filtering hand tremors, providing unmatched stability and accuracy of instrument placement. The system provides surgeons with better than 20-micron accuracy in positioning and keeping instruments stable for a long time [18]. A hybrid manual/assisted setup allows the surgeon to maintain patient contact. It places the surgeon at the top of the operating table with a robot attached to a headrest. Residual eye movements are minimized by holding the trocar and safety measures are incorporated to limit instrument movements [19,20]. Currently, it has been approved in Europe to perform procedures such as macular surgery, retinal membrane peeling, supraretinal injections, and intravenous cannulations [21,22]. The Preceyes robotic system set the record of successfully completing the world's first inocular robotic surgery by a team at John Radcliffe Hospital, Oxford [23].

Optical coherence tomography

A clear view of the eye is vital to surgery and the microscope is the widely used device for such use. However, it is sometimes difficult to visualize the intraocular region behind the endoscope due to the fixed angle of view. In addition, the condition of the cornea and eye can affect the quality of vision. these limitations are compensated with the OCT. OCT is a standard surgical procedure and diagnostic tool that develops cross-sectional images of tissue using light reflection [24]. In order to permit simultaneous imaging and surgical manipulations, microscope integration systems have been created that combine the optical pathways of the OCT and the surgical microscope. By eliminating the requirement for frequent breaks during surgery, this capability removed a significant barrier posed by a separate external big imaging probe [25]. Commercial systems are now readily accessible, with the Zeiss RESCAN 700 (Carl Zeiss AG, Oberkochen, Germany) being the first. This has made it possible to perform 3D and 4D OCT in surgery in real-time. This provides better feedback on the position of the instrument, maintaining parity and making it possible for the surgical microscope to include different axial positions, and in having various levels of zoom [26-28].

Tele-manipulation robotic systems

Real-time intraocular imaging is made possible by the combined surgical forceps and B-Mode Transition OCT probe, which increases the precision of membrane removal procedures. Stainless steel (SS) tube of 25 gauge and an SS tube of 23 gauge make up the clamp. The clamp will open and close with the outer tube sliding along the 25 gauge [28-30]. It increases precision and decreases the number of sessions needed to finish the membrane peeling operations.

Miniature robot with six degrees of freedom

This robot produces rectilinear and rotary motion using two parallel prismatic joints. the presence of parallel actuators increases overall stiffness compared to series-linked actuators [4,7,29]. it creates a very ingenious system that eliminates the surgeon's vibrations and minimizes the impact of user fatigue; however, challenges associated with intraocular maneuverability are not addressed. 

Intraocular Robotic Interventional Surgical System (IRISS)

In order to manipulate surgical tools with 6 DOFs, it has two manipulators that mount and move on independently positioned semicircular rails with two different actuators. Each instrument has a fixed RCM that is constrained by the device's shape [30]. This system's primary objective is to carry out anterior and posterior intraocular surgery procedures using automation and remote control (telemanipulation). IRISS enables surgeons to successfully carry out procedures like hydro-dissection, vitreous aspiration, retinal vein cannulation, remote-controlled anterior lens capsulorhexis, viscoelastic injection, and vitrectomy [7]. The capacity of this robot to use two surgical instruments at once is a special feature. IRISS has made history by being the first robotic system to successfully accomplish a whole circumcision and an entire cataract surgery [31].

Hand-on-hand robotic systems

They provide vibration filtering by taking advantage of the mechanical rigidity of the robotic system to control the surgical instruments at the surgeon's request. It allows the surgeon to operate an instrument positioned on a robotic mobile platform using force input controls. It also helps to recall the position and reduce the fatigue of the surgeon because the robot can keep the instrument in a stationary posture even without the surgeon holding it [32].

Steady Hand Robot and Steady Hand Robot 2

Surgeons utilise the same surgical tools as they would with conventional instruments, with the robot controller reading force signals from the surgeon's hand movements to control robot control. Robots can create smooth and natural movement records that surgeons often use in retinal procedures while removing unrelated tool movements due to vibrations [33]. When used with OCT, the stabilizing hand can maintain a defined distance between the tip of the surgical instrument and the retinal tissue to within 10 micrometers of the desired 150 micrometers [34]. Attaching a force sensor that provides the limiting force for the robot thereby restricting the peak force exerted on the intraocular tissue by providing feedback, thereby guiding the surgeon to avoid unintended destructive contact and maintain the instrument position relative to the intraocular anatomy.

Eye Robot 2 is a new method of enhanced collaborative control developed at Johns Hopkins Hospital that combines both a greatly improved controller and an integrated micro-force sensing engine [35].

Magnetically controlled robot systems

In order to perform procedures like reducing the size of the retinal veins and delivering local medication using drug-eluting microcapsules, it uses an extraocular magnetic field to guide robotic microcapsules into the eye, thereby, achieving high intraocular mobility and dexterity without a physical connection to the extraocular area. It reduces coherence between the extraocular and intraocular areas and removes any applicable movement restrictions.

Octomag

It is a magnetic field system to guide microencapsulated robots in 5 DOFs, three position control levels, and two orientation levels. Charreyon et al. utilized Octomag to manipulate a microcannula with a magnetic tip to deliver gene therapies into subrenal tissue [36,37].** **Magnetic microtechnology retains the benefits of intraocular dexterity and provides safety.

Figure 6 shows recent advances in Femtosecond Laser Filamentation [38].

**Figure 3:**
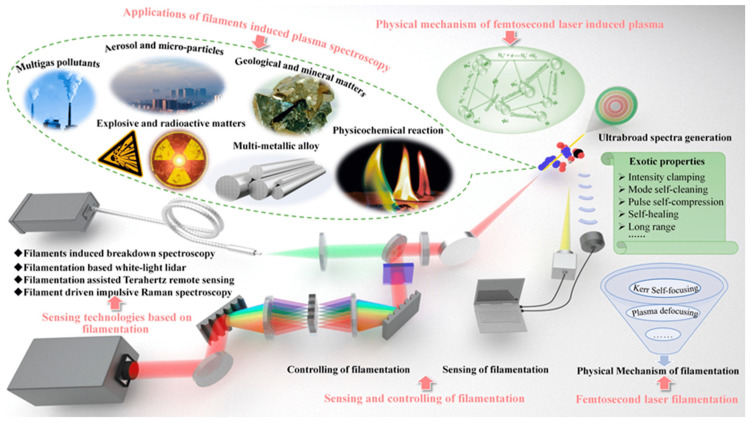
Recent advances in sensing with femtosecond laser filamentation This image has been taken from an open-access site with copyright to Qi et al. [38].

Present limitations challenges and future

Recent advertisement about Robotic Cataract Surgery in India has taken its toll on the people. A number of eye facilities in India, including AIIMS and Eye7 Hospitals in New Delhi and Medanta in Gurgaon, now provide the most advanced robotic cataract surgery employing artificial intelligence - AI LenSx Femtosecond Laser System. High-resolution anterior segment imaging and the femtosecond laser can be integrated with higher accuracy and safety using the AI LenSx Laser. This enables the surgeon to design a surgical plan using sophisticated software and a 3-D picture of the eye. So, a computer-guided laser with AI is used to execute the procedure resulting in greater accuracy, precision, and reproducibility. This platform provides better predictability forIOL (intraocular lens) power calculations and surgical incision placements resulting in almost perfect vision after cataract surgery.

Surgical robots have shown their ability and utility in advancing vitreous surgery, although they have their own limitations and challenges. Keeping the robotic tool level with the patient's head and safely and quickly retracting the tool in the event of clinical emergencies and involuntary eye movements is paramount to improving safety and effectiveness. These clinical surgical robots improve the surgeon's vision, perception, and accuracy. These robotic systems will include technologies such as OCT, with calibration methods and control algorithms that take into account safe anatomical movements during the procedure. The most common limitation in developing countries like our own India is the lack of previous experience with robotic surgeries, which may initially result in sacrificing speed for the sake of safety. The lengthened surgical procedure and related expenses are now prominent factors in robotic surgery. If the robotic system can be employed in assist mode, where it is used for precise missions and parked at a safe distance during other stages of surgery, both can be decreased. It must also be as discrete as feasible and adapt to operating rooms. To reduce costs, a robotic system should also be used. A robotic system should also be employed in numerous ophthalmic operations to cut expenses. In this sense, a remotely controlled system best satisfies these demands. It is also anticipated that some well-defined and unidentified dangers and problems will still need to be addressed given its growth and the field's relative youth. 

The happy patients with now-developed perfect vision are yet another example of how AI-based robotics are making the quality of life and professional medical assistance better. This observation shows that time and resource investments in this kind of study will pay off and argues strongly in favour of its pursuit.

## Conclusions

This article explores how the robotics have diversified and progressed with the upcoming technologies. Consierable amount of studies and trials are being conducted for various robotic systems, and only a few of them have made it to the commercial stage. The little but extremely complex field of robotic retinal surgery is developing quickly, with huge increase in knowledge and technology advancements over the past three decades. India has shown a huge acceptance to the robotic cataract surgery which has been the newest addition to the country’s ocular field. The robotics tends to cater to the demands of quality treatment with little risk to the patients and also provides a certain amount of relaxation by reducing the physical stress of the surgeons.The challenges formerly experienced in form of movement stability, precision are now addressed promoting the efficiency to perform such delicate surgeries.In light of the benefits enumerated of the robotics, continued support and investment in this field is of utmost importance. It is still in its initial stage but with increasing research and development, it will soon reach a stage where it is introduced to the people for major ocular surgeries.
